# P-1178. Antibacterial Activity of a Polygalacturonic and Caprylic Acid Wound Ointment Compared with Hypochlorous Acid Against Polymicrobial Biofilms in a Three-Dimensional Wound Biofilm Model

**DOI:** 10.1093/ofid/ofaf695.1371

**Published:** 2026-01-11

**Authors:** Bahgat Z Gerges, Joel Rosenblatt, Y Lan Truong, Ying Jiang, Issam I Raad

**Affiliations:** MD Anderson UT, Missouri City, TX; MD Anderson UT, Missouri City, TX; UT MD Anderson Cancer Center, Houston, TX; The University of Texas MD Anderson Cancer Center, Houston, Texas; MD Anderson UT, Missouri City, TX

## Abstract

**Background:**

Polymicrobial biofilms of bacteria and fungi frequently occur in chronic wounds and pose a significant barrier to healing. Polygalacturonic + caprylic acids (PG+CAP) and hypochlorous acid (HOCl) have been shown to be well tolerated yet potent antimicrobial agents for eradicating a broad range of mono-microbial biofilms in traditional biofilm models. In this study we compared their efficacies *in vitro* against polymicriobial biofilms using a three-dimensional fibrin gel wound biofilm model (FGWB).

Figure 1. Efficacies of polygalacturonic + caprylic acids (PG+CAP) and hypochlorous acid (HOCl) against mono-, and polymicrobial biofilm of methicillin resistant Staphylococcus aureus (MRSA) + Candida albicans after three-hours exposures using fibrin gel wound biofilm model (FGWB).

**Figure:**
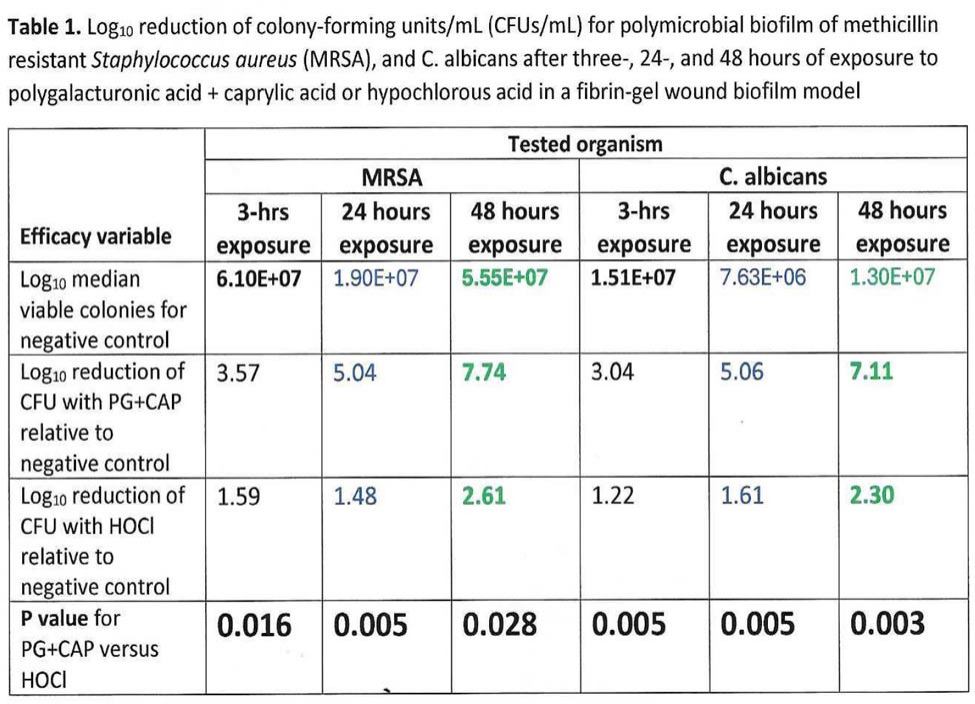

**Figure:**
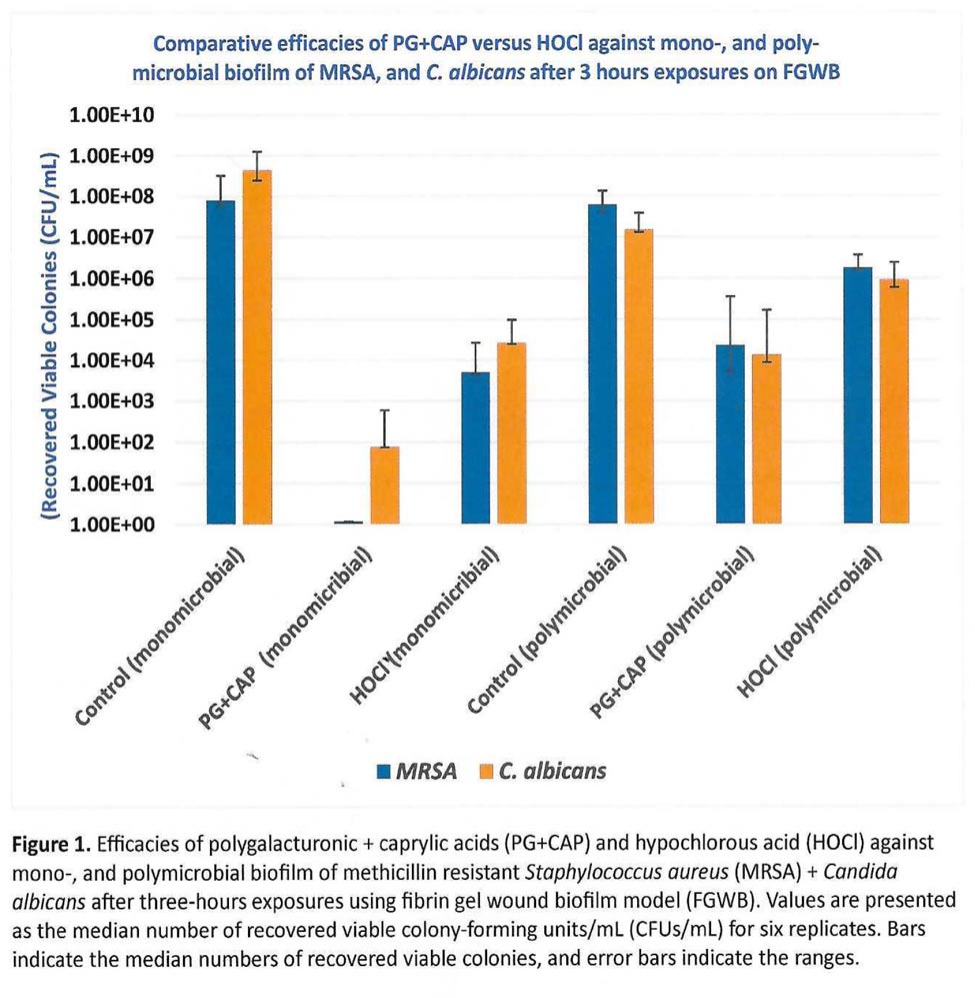

Figure 2: Efficacies of polygalacturonic + caprylic acids (PG+CAP) and hypochlorous acid (HOCl) against polymicrobial biofilm of methicillin resistant Staphylococcus aureus (MRSA)+ Candida albicans after three-, 24-, and 48-hours exposures using fibrin gel wound biofilm model (FGWB).

**Methods:**

The efficacies of PG+CAP, and HOCl were compared against a polymicrobial biofilm of MRSA + *Candida albicans* isolates in an *in vitro* FGWB model with different treatment durations. Single applications of 3 hrs, 24 hrs, and a double application of two 24 hr exposures (48 hrs total) were tested. FGWBs were formed by co-culturing the pathogens for 48 hrs in human fibrinogen solutions to which thrombin and calcium were added. Challenge exposed the FGWBs to antimicrobials for different durations following which the number of viable microbes were enumerated by sonication, serial dilution, plating and colony counting. Untreated FGWB were used as a control.

**Results:**

As shown in Figure 1, the polymicrobial biofilm showed less susceptibility to the antimicrobial agents when compared to monomicrobial biofilms. As shown in Figure 2, PG+CAP produced a greater reduction of viable organisms when compared to HOCl for all tested time exposures in the FGWB model. As shown in Table 2, the difference between PG+CAP and HOCl in eradicating both pathogens in polymicrobial biofilm was statistically significant (P≤0.05).

**Conclusion:**

Polymicrobial (MRSA + *C. albicans*) biofilm was substantially less susceptible to antimicrobial treatments than monomicrobial biofilms. PG+CAP was more effective against both mono-, and polymicrobial biofilms in the FGWB than HOCl. PG+CAP merits further *in vivo* study in settings where wounds are colonized by polymicrobial biofilms.

**Disclosures:**

Joel Rosenblatt, PhD, Citius Pharmaceuticals, Inc.: Advisor/Consultant|Citius Pharmaceuticals, Inc.: Grant/Research Support|Citius Pharmaceuticals, Inc.: Patent|Citius Pharmaceuticals, Inc.: Ownership Interest|Citius Pharmaceuticals, Inc.: Stocks/Bonds (Public Company)|Spectrum Vascular: SV Spectrum MRC Central Venous Catheter; SV Spectrum MR Central Venous Catheter; SV Central Venous Catheter|Spectrum Vascular: Ownership Interest Issam I. Raad, Distinguished Professor, Citius Pharmaceuticals, Inc. (Grant/Research Support): Advisor/Consultant|Citius Pharmaceuticals, Inc. (Grant/Research Support): Grant/Research Support|Citius Pharmaceuticals, Inc. (Grant/Research Support): Patent|Citius Pharmaceuticals, Inc. (Grant/Research Support): Ownership Interest|Citius Pharmaceuticals, Inc. (Grant/Research Support): Stocks/Bonds (Public Company)|Spectrum Vascular: Patent|Spectrum Vascular: Ownership Interest

